# Inheritance and QTL mapping of cucumber mosaic virus resistance in cucumber (*Cucumis Sativus* L.)

**DOI:** 10.1371/journal.pone.0200571

**Published:** 2018-07-18

**Authors:** Lixue Shi, Yuhong Yang, Qing Xie, Han Miao, Kailiang Bo, Zichao Song, Ye Wang, Bingyan Xie, Shengping Zhang, Xingfang Gu

**Affiliations:** Institute of Vegetables and Flowers, Chinese Academy of Agricultural Science, Beijing, China; College of Agricultural Sciences, UNITED STATES

## Abstract

The commercial yield of cucurbit crops infected with Cucumber mosaic virus (CMV) severely decreases. Chemical treatments against CMV are not effective; therefore, genetic resistance is considered the primary line of defense. Here, we studied resistance to CMV in cucumber inbred line ‘02245’ using a recombinant inbred line (RIL) population generated from a cross between ‘65G’ and ‘02245’ as susceptible and resistant parents, respectively. Genetic analysis revealed that CMV resistance in cucumber is quantitatively inherited. Analysis of the RIL population revealed that a quantitative trait locus (QTL) was found on chromosome 6; named *cmv6*.*1*, this QTL was delimited by SSR9-56 and SSR11-177 and explained 31.7% of the phenotypic variation in 2016 and 28.2% in 2017. The marker SSR11-1, which is close to the locus, was tested on 78 different cucumber accessions and found to have an accuracy of 94% in resistant and moderately resistant lines but only 67% in susceptible lines. The mapped QTL was delimited within a region of 1,624.0 kb, and nine genes related to disease resistance were identified. Cloning and alignment of the genomic sequences of these nine genes between ‘65G’ and ‘02245’ revealed that Csa6M133680 had four single-base substitutions within the coding sequences (CDSs) and two single-base substitutions in its 3’-untranslated region, and the other eight genes showed 100% nucleotide sequence identity in their exons. Expression pattern analyses of Csa6M133680 in ‘65G’ and ‘02245’ revealed that the expression levels of Csa6M133680 significantly differed between ‘65G’ and ‘02245’ at 80 h after inoculation with CMV and that the expression in ‘02245’ was 4.4 times greater than that in ‘65G’. The above results provide insights into the fine mapping and marker-assisted selection in cucumber breeding for CMV resistance.

## Introduction

Cucumber mosaic virus (CMV) is a plant pathogenic virus of the genus *Cucumovirus* in the family *Bromoviridae* [1]. It has a worldwide distribution and a very broad range of hosts, and it was first reported in 1934 in cucumbers that showed mosaic symptoms [2]. Throughout their whole growth cycle, cucumber crops can be damaged by CMV; damaged plants exhibit symptoms that include strong leaf mosaic patterns and leaf distortion, stunted growth, reduced flower production, and fruit lesions, all of which overall result in yield reductions of 10–20% [3]. Because this virus has a broad host range and many insect vectors and because it cannot be controlled by chemicals, the most likely method of control is by genetic resistance [4].

Reports of genetic resistance to CMV are inconsistent and include dominant, recessive, monogenic, and polygenic controls. Resistance in *Arabidopsis thaliana* ecotype C-24 and common bean was reported to be controlled by monogenic dominant genes [5–6], whereas in chili pepper (*Capsicum annuum* L.), resistance is quantitatively inherited [7–8]. Zhou reported that resistance in squash is due to a major gene as well as a recessive gene [9], but Celai reported that resistance in melon accession ‘PI161375’ is governed by one gene and at least two quantitative trait loci (QTLs) [10]. Among cucurbits, resistance to CMV is believed to be mostly recessive [11]. Reports about the inheritance of CMV resistance in cucumber are not consistent due to the effects of different sources of the virus and different experimental designs and methods. Wasuwat reported that resistance in the cultivar Wis. SMR 12 is controlled by a single dominant gene, whereas Kooistra suggested that resistance was due to three independent dominant genes [12–13]. On the other hand, Wang, Huang, and Munshi reported that resistance is controlled by a single recessive gene [14–16].

Few studies have investigated the molecular mechanism of resistance to CMV in cucumber. Huang used an F2 population derived from cucumber lines ‘HZL04-1’ (susceptible) and ‘F-3’ (resistant) and found a dominant 204 bp long amplified fragment length polymorphism (AFLP) marker that was tightly linked to the CMV resistance gene at a genetic distance of 8.57 cM and transformed into a sequence characterized amplified region (SCAR) marker, *cmv-*200 [15]. Wang identified seventeen QTLs that control resistance in ‘F-3’ lines; these QTLs were named *cmv*1 through 17. *cmv17* was determined to be a primary locus that controls resistance to CMV, and this locus explained 67.3% of the variance. Furthermore, this locus was closely linked to the cucumber anti-CMV SCAR marker *cmv-*200 at a distance of only 0.5 cM [17].

In the present study, a recombinant inbred line (RIL) derived from a cross between the susceptible ‘65G’ and resistant ‘02245’ cucumber inbred lines was artificially inoculated with CMV, and used to identify QTLs and candidate genes linked to CMV resistance.

## Materials and methods

### Plant materials and population development

Cucumber inbred lines ‘65G’ (susceptible) and ‘02245’ (resistant) were provided by the Department of Cucurbits Genetics and Breeding, Institute of Vegetable and Flowers, Chinese Academy of Agricultural Science, Beijing, and used as parental lines to develop the F_1_ and RIL (140 lines) populations. The RIL population was developed by single seed descent.

### Inoculations and virus detection

The CMV isolate was collected from the leaves of infected tobacco plants provided by the Department of Plant Protection, Institute of Vegetables and Flowers, Chinese Academy of Agricultural Sciences, Beijing. One gram of fresh leaf tissue was ground in 10 mL of 0.03 M phosphate buffer (pH 7.0), which was subsequently rubbed into the first fully expanded true leaf of cucumber seedlings that were dusted with carborundum powder prior to inoculation. The leaves were rinsed with water, and the inoculation was repeated 2–3 days later [18]. The RIL population was organized into a randomized complete block with three replicates, with each replicate containing seven plants, and maintained in a greenhouse at 25–28°C under natural sunlight.

**Fig 1:**
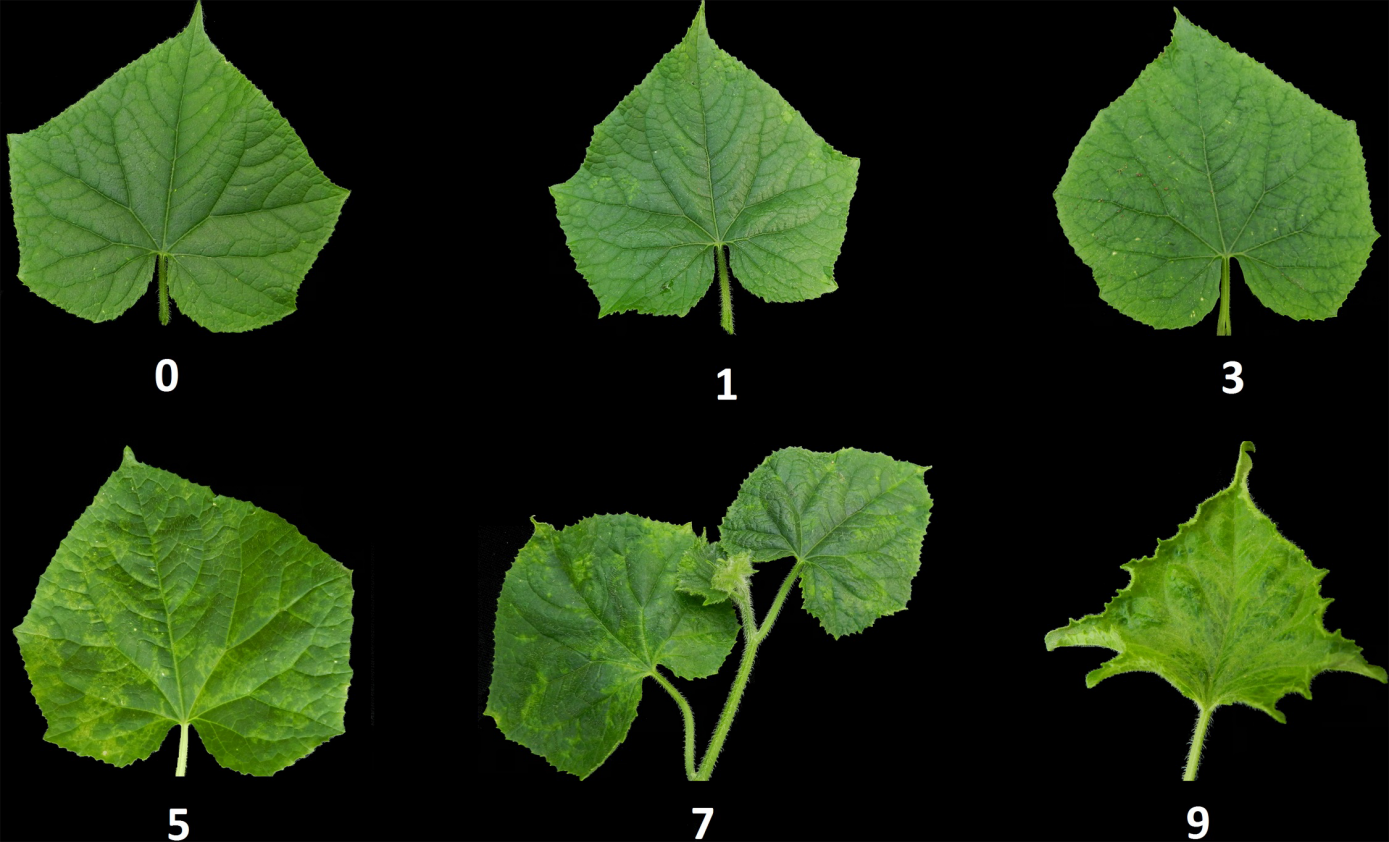
The 0–9 infection ranking system was based on the severity of mosaic patterning and leaf distortion. 0 = no symptoms; 1 = apical leaves with dispersed vein clearing or slight mottling; 3 = mosaic patches and/or necrotic spots on leaves; 5 = moderate mosaic patches and moderate distortion of the three youngest leaves; 7 = apical meristem exhibiting mosaic patches and deformation; 9 = extensive leaf mosaic patches and severe distortion of all leaves—even plant death.

The seedlings were observed at 20 days after inoculation and rated in accordance with a 0–9 infection-type ranking system described by Tian [19]. Symptoms of the CMV are shown in Fig 1. Disease indices (DI) for the RIL population were calculated from the ranking system by the formula:

DI = ∑[(s×n)/(S×N)]×100% [20]

(s: disease grade, n: number of plants in the disease grade, S: highest disease rating scale, N: total numbers of plants).

A double-antibody sandwich enzyme-linked immunosorbent assay (DAS-ELISA) was used to determine the virus concentration in the apical leaves at 20 days after inoculation [21]. The CMV-specific kit was purchased from Agdia, Elkhart, IN, USA.

### Linkage map construction

The 140 lines of the RIL population were used to construct a linkage map. Simple sequence repeat (SSR) markers were obtained from the genome of cucumber inbred line ‘9930’ [22]. A total of 1,228 SSR primers distributed across all seven chromosomes of cucumber were used to screen for polymorphisms in the parents. Primers that showed polymorphisms were used to construct the genetic linkage map.

Genomic DNA was extracted from young cucumber leaves using the modified cetyl-trimethylammonium bromide (CTAB) method reported by Wang [23]. PCR amplification and agarose gel electrophoresis were performed as described by Song [24]. Linkage map construction via polymorphic markers was performed using JoinMap^®^ 4.0, where the band pattern of the maternal parent is denoted as a, the band pattern of the paternal parent is denoted as b, and the heterozygous band as h [25].

### Inheritance analysis and QTL mapping

Statistical calculations were performed by Microsoft^®^ Excel 2007. QTL analysis was performed via the interval mapping (IM) and multiple-QTL model (MQM) methods with MapQTL^®^ 4.0 (Kyazma B.V., Wageningen, Netherlands). The detected locus was named in accordance with the following scheme: the abbreviation of CMV, chromosome (Chr.) number, and locus number.

### Validation of SSR markers linked to the CMV resistance gene

The authenticity of markers that were closely linked to the CMV resistance gene was tested by the inheritance data of 78 different cucumber inbred lines and hybrids.

### Prediction and molecular cloning of the candidate gene

The candidate genes for CMV resistance were identified by comparison with the reference genome of cucumber ‘9930’ (http://cucurbitgenomics.org/). Gene function was predicted by BLAST based on sequence alignment with sequences in the SwissProt and Gene Ontology (GO) databases. Specific primers were designed to amplify the candidate gene sequences between ‘65G’ and ‘02245’. PCR amplification was performed in a total volume of 25 μL with KOD FX (Toyobo), and the PCR products were sequenced by Sangon Biotech (Beijing).

### Extraction of nucleic acids and qRT‑PCR

Young developed cucumber leaves (0 h, 16 h, 40 h, 80 h, 132 h and 192 h after inoculation with CMV) were collected from both parents and were flash frozen in liquid nitrogen; three biological replications of each sample were included. The total RNA was extracted using an RNeasy Plant Mini Kit (TaKaRa 9769) and a PrimeScript^TM^ Reagent Kit with gDNA Eraser (TaKaRa). qRT-PCR was conducted using SYBR Premix Ex Taq^TM^ II (TaKaRa), and PCR amplification was performed using a qPCR instrument. *Actin1* was used as a reference gene for normalizing gene expression values. The analysis of candidate gene relative expression data was performed using the 2^−ΔΔCt^ method.

## Results

### Inheritance of CMV resistance in cucumber

**Fig 2:**
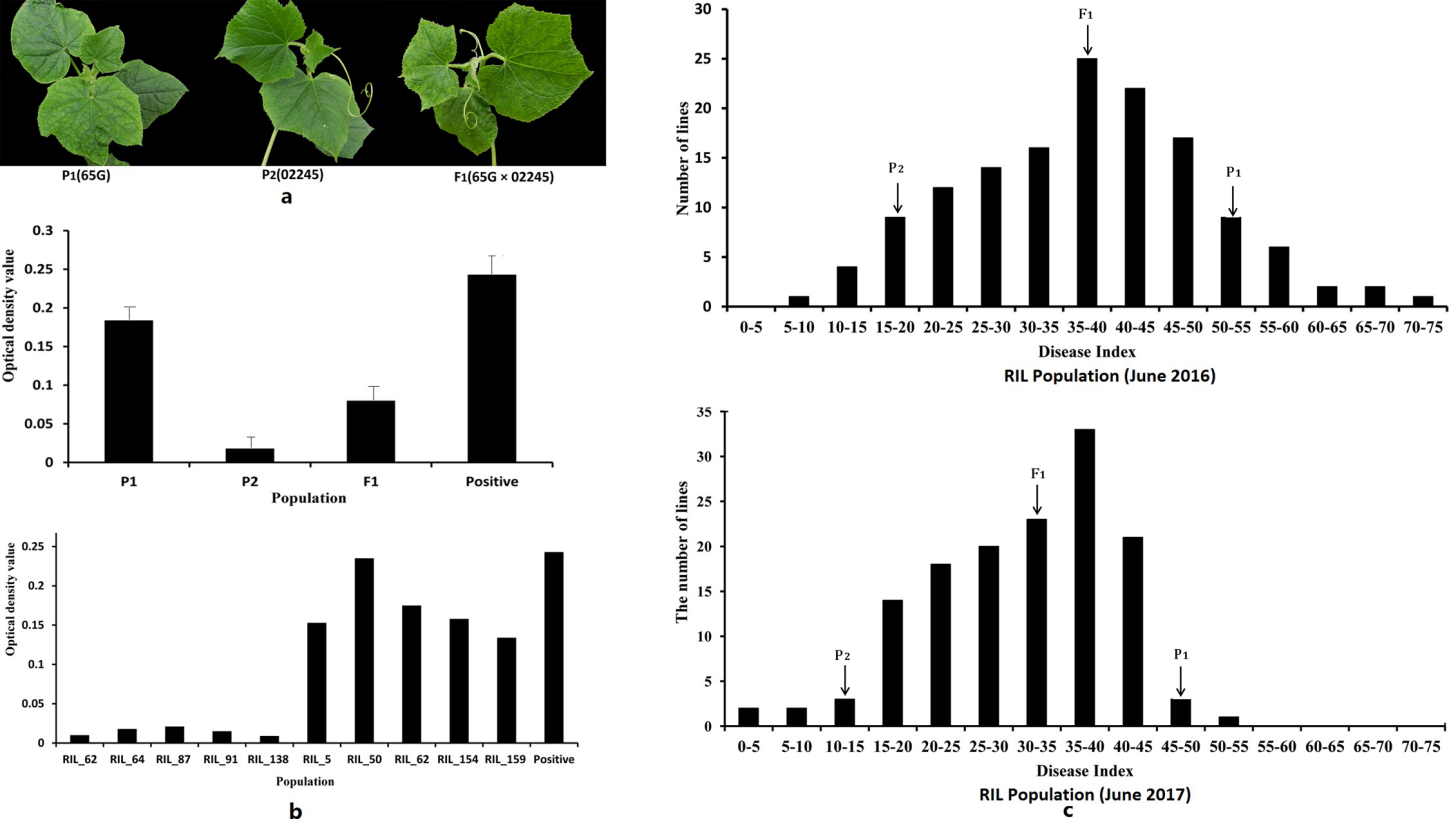
ELISA results for CMV and the distribution of CMV DIs. a Symptoms of the susceptible parental line ‘65G’, the resistant line ‘02245’ and their F_1_ hybrid progeny after they were inoculated with CMV. b DAS-ELISA results for CMV in the leaves of P1 (‘65G’), P2 (‘02245’) and F1 plants as well as some plants within the RIL population; the result is consistent with the phenotypic identification. c Frequency distribution of the DI from CMV among the ‘65G’×‘02245’ RIL population. The frequency distribution in June 2016 and June 2017 each presented a normal distribution ranging from resistant to susceptible phenotypes.

The resistant parental line, ‘02245’, had a DI of 15.75 in 2016 and a DI of 11.1 in 2017 and exhibited none of the symptoms of CMV at 20 days after inoculation; however, the susceptible parental line, ‘65G’, had a DI of 50.43 in 2016 and a DI of 48.14 in 2017 and showed severe mosaic patterning and leaf distortion. The F_1_ hybrids had a DI of 35.80 in 2016 and a DI of 34.72 in 2017 and exhibited symptoms that were more similar to the susceptible parent (‘65G’) than to the resistant parent (‘02245’) (Table 1 and Fig 2A). For the RIL population, the DI values showed a continuous and near-normal distribution, suggesting that resistance to CMV in cucumber is quantitatively inherited (Fig 2C). The results for the DAS-ELISA tests confirmed the efficiency of the inoculations (Fig 2B).

**Table 1:** The DIs of the parental lines and the F_1_ and RIL populations.

Environment	Parental Lines (Mean±S.E)		F_1_ (Mean±SE)	RIL Population			
	P1 (‘65G’)	P2 (‘02245’)		Mean±SE.	SD^a^	Skewness	Kurtosis
**June 2016**	50.43±0.69	15.75±0.63	35.80±0.35	37.33±1.10	13.09	0.11	-0.19
**June 2017**	48.14±0.25	11.11±0.00	34.72±0.12	30.76±0.81	9.67	-0.51	-0.18

^a^SD: standard deviation

### Linkage map construction

Of the total 1,228 SSR primers used to screen for polymorphisms within the parental lines, 296 (22.98%) were found to be polymorphic. Primers that were evenly distributed across the seven chromosomes were selected for constructing the linkage groups. Ninety-seven primers were selected for the RIL population; these markers were mapped onto seven linkage groups that covered 730.0 cM. The average distance between the markers was 7.5 cM, and each linkage group was distributed across approximately 8–32 markers (S1 Table and S1 Fig). The seven linkage groups in RIL population were assigned to cucumber chromosomes based on the SSR marker data and publicly available cucumber map data.

### QTL mapping and analysis

**Fig 3:**
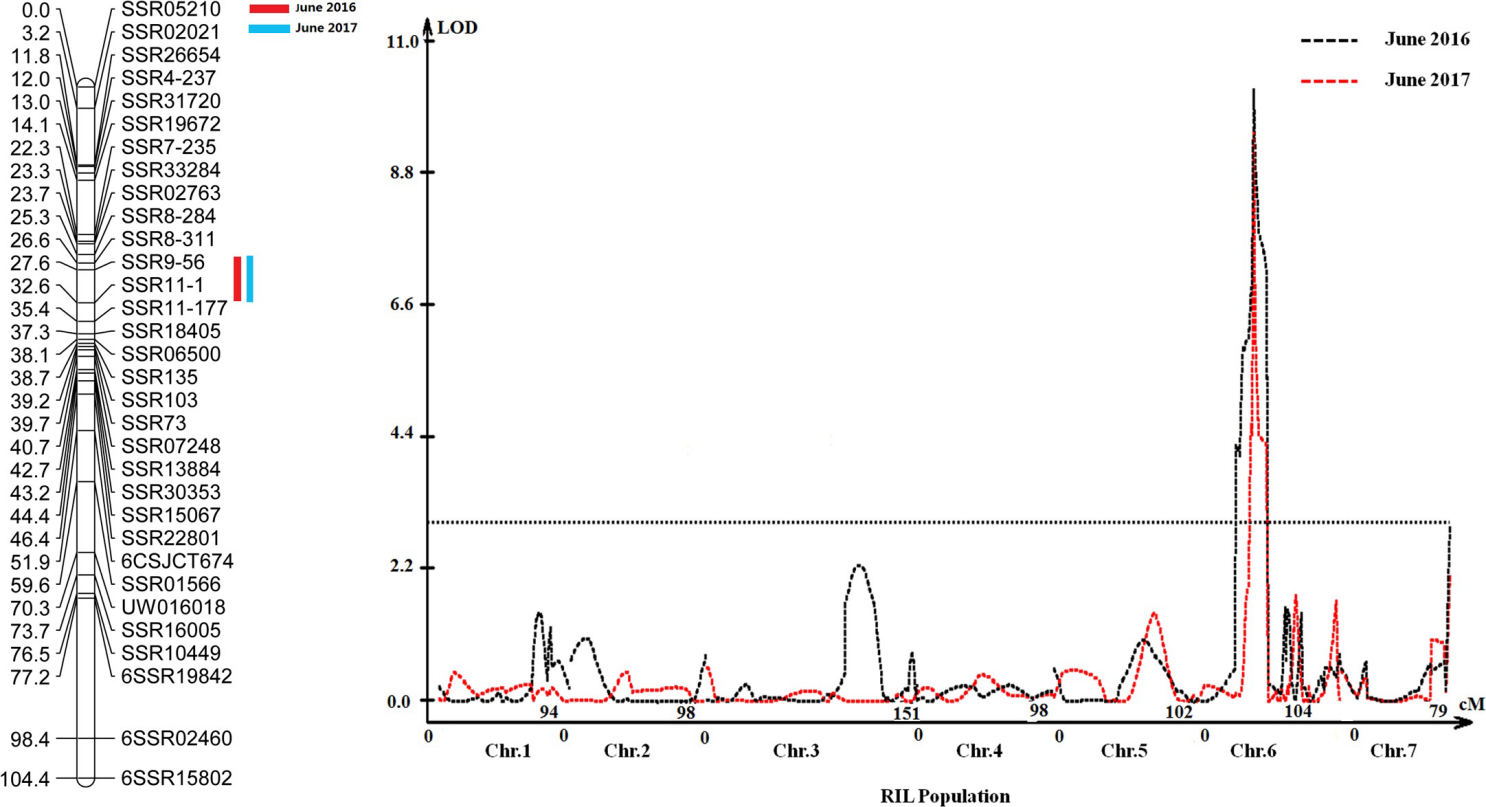
QTL controlling CMV resistance and its effect on cucumber seedlings during two years. One QTL was detected at the same location in June 2016 and June 2017; this QTL was identified on chromosome 6 (Chr.6). Note: The red box indicates June 2016, the blue box indicates June 2017, the black line indicates June 2016, and the red line indicates June 2017.

The genetic maps of the RIL population were used to detect QTLs for resistance to CMV. IM via MapQTL^®^ 4.0 identified a QTL for CMV on chromosome 6 (linkage group 6) in both years, which was named *cmv6*.*1*. The QTL for CMV was located within 32.7 cM on chromosome 6 and was delimited by two SSR markers, SSR9-56 and SSR11-177. The logarithm of odds (LOD) scores in 2016 and 2017 were 11.58 and 10.09, respectively, and accounted for 31.7% and 28.2% of the phenotypic variation, respectively (Table 2 and Fig 3).

**Table 2:** QTL controlling CMV resistance and its effect on cucumber seedlings.

Environment	Population	QTL	Chromosome	Marker Interval	Peak Location	LOD	R^2^ (%)	AE^a^
**June 2016**	RIL	*cmv6*.*1*	Chr.6	SSR9-56–SSR11-177	32.7 cM	11.58	31.7	7.39
**June 2017**	RIL	*cmv6*.*1*	Chr.6	SSR9-56–SSR11-177	32.7 cM	10.09	28.2	5.15

^a^ AE: Additive effects

### Validation in molecular markers linked to the *cmv6*.*1* locus for marker-assisted selection (MAS)-based breeding

Marker SSR11-1, the closest to the *cmv6*.*1* locus, was tested on 78 cucumber inbred lines and hybrids, of which 48 were resistant, 21 were moderately resistant, and nine were susceptible. For three of the susceptible lines (15, 25 and 73), the genotype and phenotype were inconsistent. For three lines that were either resistant or moderately resistant (12, 58 and 59), the genotype did not match the phenotype. Thus, marker SSR11-1 is 94% accurate in resistant or moderately resistant lines but 67% accurate in susceptible lines (S2 Table).

### Prediction and molecular cloning of the candidate gene

**Fig 4:**
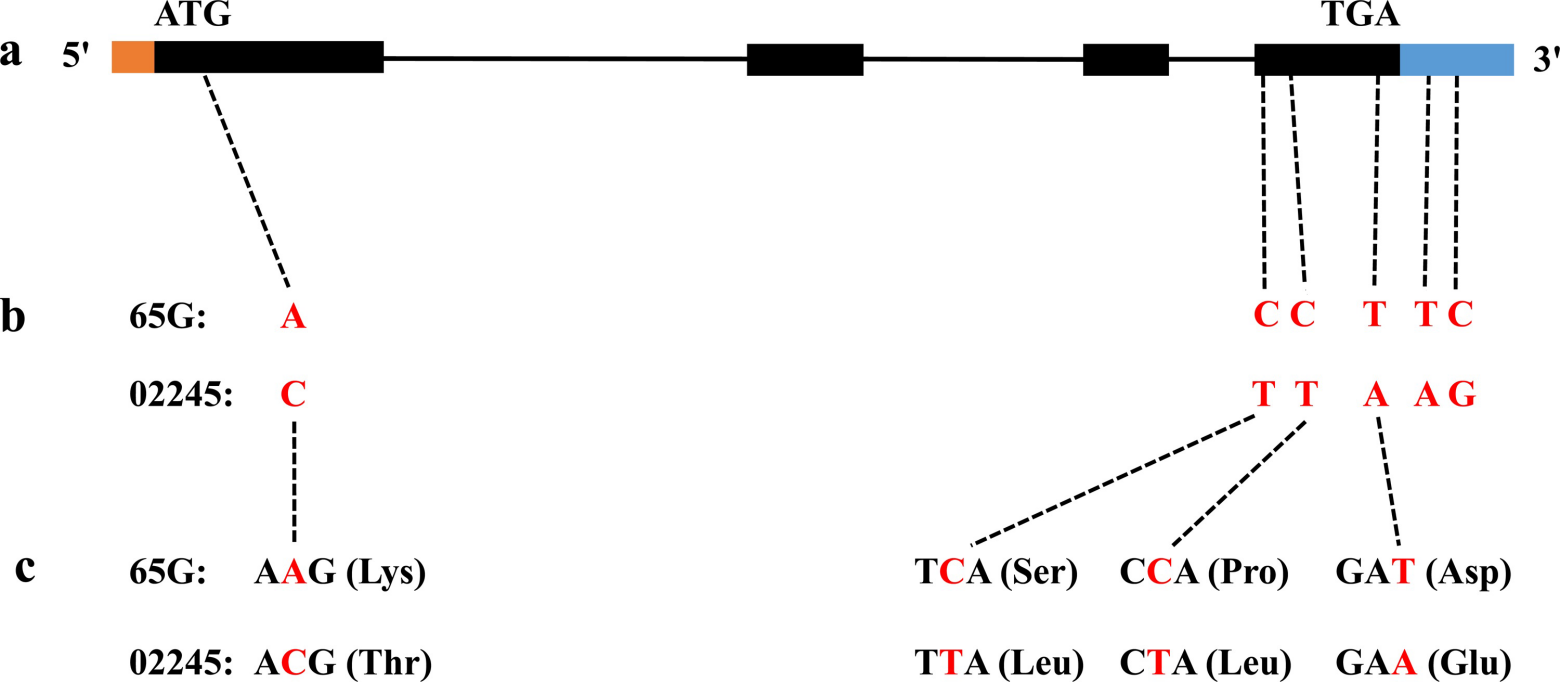
Mutation of the Csa6M133680 gene. **a Distribution of 4 exons and 3 introns within the coding region of Csa6M133680.** b A single-nucleotide substitution (A / C) in exon 1, three single-nucleotide substitutions (C / T, C / T, T / A) in exon 4 and two single-nucleotide substitutions (T / A, C / G) in the 3’-untranslated region. c The four single-nucleotide substitution caused four amino acid substitutions (Lys / Thr, Ser / Leu, Pro / Leu, Asp / Glu). Note: The orange box indicates the 5’-untranslated region, the black box indicates coding regions, the black line indicates an intron, and the blue box indicates the 5’-untranslated region.

The mapped QTL was found to be delimited within a region of 1,624.0 kb on chromosome 6, and after aligning with data from the Cucurbit Genomics Database (ICUGI, http://www.icugi.org/), 151 genes were predicted. Among these genes, nine related to disease resistance were identified. The predicted functions and associated information of these nine resistance genes are presented in Table 3. We amplified the genomic sequences of these nine genes within both ‘65G’ and ‘02245’. Sequencing and aligning of the genomic sequences indicated that the Csa6M133680 sequence had four single-base substitutions within the coding sequences (CDSs) and two single-base substitutions in the 3’-untranslated region between ‘65G’ and ‘02245’ (Fig 4A), and the other eight genes showed 100% nucleotide sequence identity in the exons between ‘65G’ and ‘02245’. Gene annotation revealed that Csa6M133680 had four exons and three introns, all of which encoded a predicted protein of 397 amino acids. The single-base substitutions caused an amino acid substitution in exons 1 and 4 (Fig 4B and 4C). The functional annotation of Csa6M133680 revealed that the gene product is a basic leucine zipper (bZIP) domain-containing transcriptional activator.

**Table 3:** Results of CMV resistance gene predictions in cucumber.

Gene ID	Position on Chr.6	Predicated Gene Function
**Csa6M117180**	8019818–8020225	RING finger-like; contains IPR013083 (zinc finger, RING/FYVE/PHD-type)
**Csa6M118880**	8231204–8234737	U-box domain-containing protein; contains IPR013083 (zinc finger, RING/FYVE/PHD-type) and IPR016024 (armadillo-type fold)
**Csa6M124180**	8624484–8626721	Ethylene-responsive transcription factor 5; contains IPR016177 (DNA-binding, integrase-type)
**Csa6M124200**	8634332–8635995	F-box/leucine-rich repeat (LRR)-repeat protein; contains IPR001810 (F-box domain, cyclin-like) and IPR006566 (FBD)
**Csa6M125260**	8697723–8700363	F-box family protein; contains IPR001810 (F-box domain, cyclin-like) and IPR005174 (protein of unknown function DUF295)
**Csa6M127350**	8816081–8820672	bZIP-related transcription factor-like; contains IPR006867 (domain of unknown function DUF632) and IPR006868 (domain of unknown function DUF630)
**Csa6M128040**	8991216–8993879	RING finger-containing E3 ubiquitin ligase; contains IPR013083 (zinc finger, RING/FYVE/PHD-type)
**Csa6M133680**	9153236–9156966	bZIP transcription factor family protein; contains IPR004827 (bZIP domain)
**Csa6M133750**	9234605–9239095	Putative receptor-like protein kinase; contains IPR011009 (protein kinase-like domain), IPR013210 (LRR-containing N-terminal, type 2), and IPR013320 (concanavalin A-like lectin/glucanase, subgroup)
**Csa6M133770**	9243958–9245770	Ethylene-responsive transcription factor 1A; contains IPR016177 (DNA-binding, integrase-type)

### Gene expression pattern analysis

**Fig 5:**
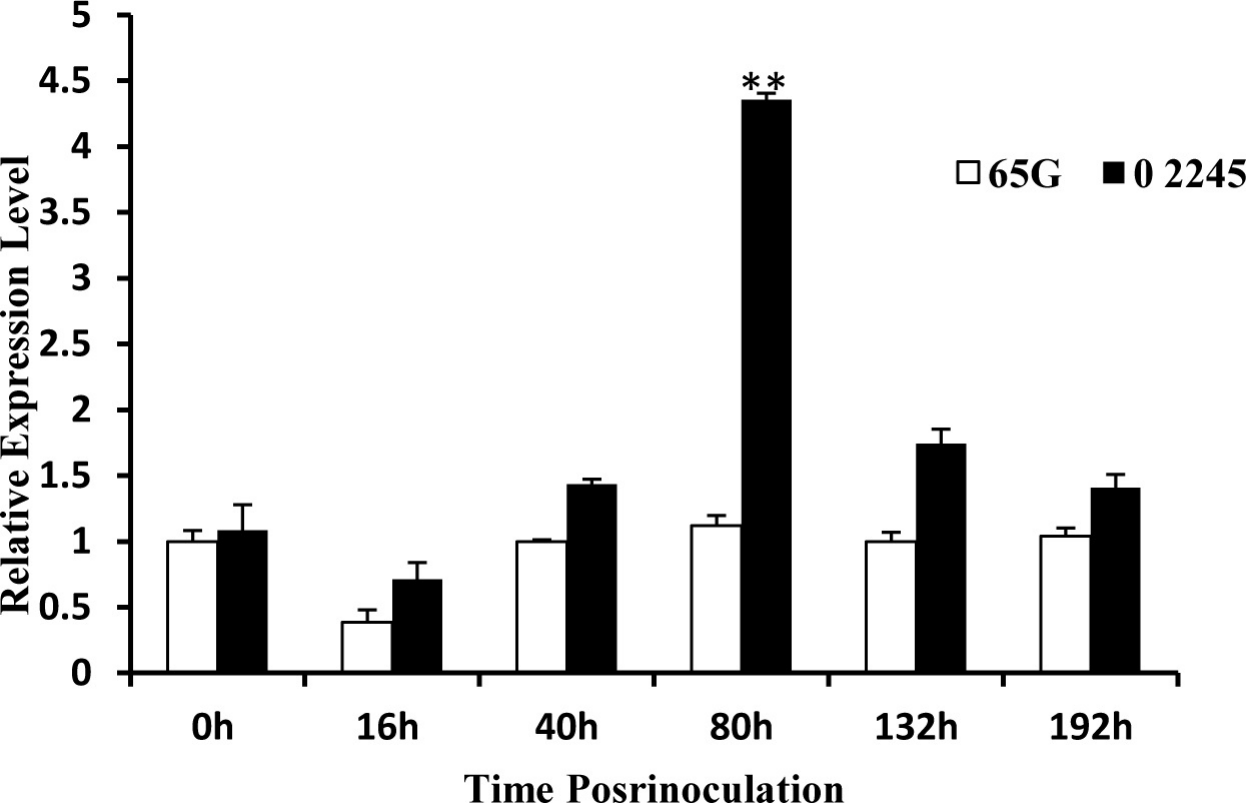
Relative quantitative expression analysis of Csa6M133680 in ‘65G’ and ‘02245’. The data represent the expression of Csa6M133680 relative to that of actin, as measured by qRT-PCR at different times after inoculation of both ‘65G’ and ‘02245’. Note: The error bars represent the standard error of three biological replicates. **Significant difference (P<0.01). The six treatments (0 h,16 h, 40 h, 80 h, 132 h and 192 h after inoculation with CMV) of ‘65G’ and ‘02245’ corresponds to the six columns in the histogram.

The total RNA that was extracted from cucumber leaves at different inoculation times (0 h, 16 h, 40 h, 80 h, 132 h and 192 h after inoculation with CMV) was used to examine the temporal expression patterns of Csa6M133680. The results showed that the expression level of Csa6M133680 significantly differed between ‘65G’ and ‘02245’ at 80 h after inoculation with CMV; in addition, the expression of Csa6M133680 in ‘02245’ was enhanced by CMV inoculation and was 4.4 times greater than that in ‘65G’ (Fig 5). Therefore, Csa6M133680 may be involved in CMV resistance regulation in cucumber.

## Discussion

### Inheritance of and QTLs for CMV resistance in cucumber

This study used the RIL population derived from a cross between cucumber inbred lines ‘65G’ (susceptible) and ‘02245’ (resistant) to determine the genetic basis for resistance to CMV. The DIs calculated for this segregating population were continuously distributed, which was appropriate for a quantitative inheritance model. These results are consistent with those of Kooistra and Wang [13] but different from those of Wasuwat, Wang, and Huang [12,14,15]. Previous studies on CMV resistance in cucumber have shown inconsistent results with respect to patterns of inheritance due to different sources of the virus and different experimental designs and methods. In the present study, methods for the identification of disease symptoms were carried out in accordance with the criteria published by the Ministry of Agriculture of the People’s Republic of China (2010). In addition, the use of DAS-ELISAs suitable for the detection of viruses of cucurbitaceous plants confirmed the infection methods used and therefore increased the accuracy of the identification of disease symptoms [26]. A QTL analysis of the RIL population was used to produce a map that covered seven linkage groups spanning a distance of 730.0 cM; the average distance between the markers was 7.5 cM (S1 Fig). This genetic map was dense enough to allow identification of QTLs for CMV resistance in cucumber.

Wang detected seventeen QTLs that control CMV resistance in a cucumber F_2_ population. These QTLs were named *cmv1* through *17* and were located on chromosomes 3, 4, 5, and 6. *cmv1* was mapped to chromosome 3, *cmv2–cmv7* were mapped to chromosome 4, and *cmv15–cmv17* to chromosome 5, the last of which had the largest phenotypic variation. *cmv8–cmv14*, which were mapped to chromosome 6, were located between markers SSRc68 and SSR20218 (15,063,719 to 15,498,366 bp) [17]. In this study, one QTL detected in the RIL population was mapped to chromosome 6, defined by markers SSR9-56 and SSR11-177 (8,004,868 to 9,628,895 bp).

### Virus resistance genes in plants

Presently, ten virus resistance genes have been cloned from several plant cultivars [27]. These genes include *RCY1*, which is related to resistance to CMV in *Arabidopsis thaliana* [5], encodes a CC-NB-LRR-class protein, and causes a hypersensitive response to CMV; the N resistance gene from Tobacco mosaic virus (TMV) in tobacco [1, 28, 29]; the *Rx* and *Rx2* genes, which provide resistance to potato virus X (PVX); the *Y-1* gene, which provides resistance to potato virus Y (PVY) in potato; the *Tm-2*^*2*^ gene, which provides resistance to Tomato mosaic virus (ToMV); and the *Tm-2* gene, which provides resistance to TMV in tomato.

In the present study, nine genes were related to disease resistance within the *cmv* mapping region. Cloning and alignment of these nine genes between ‘65G’ and ‘02245’ revealed that Csa6M133680 had multiple single-base substitutions and that the Csa6M133680 gene product is a bZIP domain-containing transcriptional activator that is homologous to the gene product of RF2b in rice, which inhibits replication by the rice tungro bacilliform virus (RTBV) (Table 3).

To further determine the possible candidate gene, we conducted qRT-PCR to analyze the temporal expression pattern of Csa6M133680 at different times after inoculation of the two parents. The results showed that the expression level of Csa6M133680 in ‘02245’ significantly increased at 80 h after inoculation with CMV and was 4.4 times greater than that in ‘65G’. Therefore, Csa6M133680 may be involved in CMV resistance regulation in cucumber, and additional fine mapping within the region delimited for CMV resistance will narrow the mapping region for the location of the resistance gene and help elucidate the mechanism of disease resistance.

### Comparison and analyses of WMV, PRSV, ZYMV and CMV resistance genes in cucumber

Viruses are the most important diseases of cucumber, and Watermelon mosaic virus (WMV), Papaya ringspot virus (PRSV), Zucchini yellow mosaic virus (ZYMV) and CMV are the four main cucumber viruses. Genes involved in resistance to WMV, PRSV and ZYMV have been mapped to chromosome 6 [30–32]. S2 Fig shows a comparison of the integrated map for resistance genes compared with that for CMV. The proximity of the genetic distances between these resistance genes is further evidence that virus resistance genes in cucumber are located within the same gene cluster.

## Supporting information

S1 Fig Construction and QTL Mapping for cucumber mosaic virus (CMV) resistance in a cucumber RIL Population using SSR markers. (DOCX)

S2 Fig Integrated mapping of resistance genes to CMV, WMV, PRSV, and ZYMV in cucumber. (DOCX)

S1 Table Information of 97 SSR markers placed on the 65G×02245 linkage map. (DOCX)

S2 Table Validity of the marker SSR11-1 tightly linked with *cmv* in 78 cucumber materials. Marker genotype designation: a, 65G allele (*CMV*^*02245*^/*CMV*^*02245*^); b, 02245 allele (*cmv*^*02245*^/*cmv*^*02245*^).
(DOCX)

## References

[1] Edwardson J R, Christie R G. CRC handbook of viruses infecting legumes. CRC Press Inc 1992; 505.

[2] Price W C. Isolation and study of some yellow strains of cucumber mosaic. Phytopathology. 1934; 24:743–761.

[3] Pan JQ. Comprehensive prevention and control technology of cucurbits virus disease. Journal of Shanghai Vegetables. 2009; (4): 86 (in Chinese).

[4] Lin CY, Ku HM, Chiang YH, Ho HY, Yu TA, Jan FJ. Development of transgenic watermelon resistant to Cucumber mosaic virus and Watermelon mosaic virus by using a single chimeric transgene construct. Transgenic Res. 2012; 21(5):983–993. 10.1007/s11248-011-9585-8 22203520

[5] Takahashi H, Miller J, Nozaki Y, Takeda M, Shah J, Hase S, et al RCY1, an Arabidopsis thaliana RPP8/HRT family resistance gene, conferring resistance to cucumber mosaic virus requires salicylic acid, ethylene and a novel signal transduction mechanism. Plant J. 2002; 32:655–667. 12472683 10.1046/j.1365-313x.2002.01453.x

[6] Seo Y S, Rojas M, Lee J Y, Lee S W, Jeon J S, Ronald P, et al A viral resistance gene from common bean functions across plant families and is up-regulated in a non-virus-specific manner. Proc Natl Acad Sci USA. 2006; 103:11856–11861. 10.1073/pnas.0604815103 16880399 PMC1567666

[7] Yao MH, Li N, Wang F, Ye ZB. Genetic analysis and identification of QTLs for resistance to cucumber mosaic virusin chilipepper(Capsicum annuum L.). Euphytica. 2013; 193:135–145.

[8] Guo GJ, Wang SB, Liu JB, Pan BG, Diao WP, Ge W, et al Rapid identification of QTLs underlying resistance to Cucumber mosaic virusin pepper (Capsicum frutescens). Theor Appl Genet. 2016; 10.1007/s00122-016-2790-3 27650192

[9] Zhou H. Identification of RAPD molecular markers linked to CMV resistance gene in squash. Dissertation, Xinjiang Agricultural University. 2005.

[10] Cèlia G A, Antonio J M, Montserrat S, Ronan X C, Jordi G M, Ana M H. The complex resistance to cucumber mosaic cucumovirus (CMV) in the melon accession PI161375 is governed by one gene and at least two quantitative trait loci. Molecular Breeding. 2014; 34(2): 351–362.

[11] Risser G, Pitrat M, Rode J C. Etude de la résistance du melon (Cucumis melo L.) au virus de la mosaïque du concombre. Ann Amél Plant. 1977; 27:509–522.

[12] Wasuwat S L, Walker J C. Inheritance of resistance in cucumber to cucumber mosaic virus. Phytopathology. 1961; 51: 423–428.

[13] Kooistra E. Inheritance of resistance cucumis virus in cucumber (cucumis sativus L.). Euphytica. 1969; 18: 326–332.

[14] Wang HJ, Wu Y, Gu W, Sun XD, Qin ZW. Extraction of DNA from cucumber by improved CTAB method. Hei-longjiang Agric Sci. 2006; 5:124–125.

[15] Huang H H. Studies on molecular maker of cucumber mosaic virus resistance-related gene in cucumber (cucumis sativus L.) Dissertation, Northwest A&F University. 2007.

[16] Marathe R, Guan Z, Anandalakshmi R, Zhao H, Dinesh-Kumar S P. Study of Arabidopsis thaliana resistome in response to Cucumber mosaic virus infection using whole genome microarray. Plant Mol. Biol. 2004; 55:501–520. 10.1007/s11103-004-0439-0 15604696

[17] Wang J H. Construction of a genetic linkage map and QTL analysis of the anti-CMV trait in cucumber (cucumis sativus L.). Dissertation, Northwest A&F University. 2010.

[18] Ministry of Agriculture of the People’s Republic of China. Rules for valuation of cucumber for resistance to diseases. 2010. e Part 7: rule for evaluation of cucumber for resistance to Cucumber mosaic virus(NY/T 1857.7–2010 Beijing)

[19] Tian GL, Miao H, Yang YH, Zhou J, Lu HW, Wang Y, et al Genetic analysis and fine mapping ofWatermelon mosaic virus resistance gene in cucumber. Mol Breeding. 2016; 36:131.

[20] Zhang SP, Miao H, Gu XF, Yang YH, Xie BY, Wang XW, et al Genetic mapping of the scab resistance gene in cucumber. J Am Soc Hortic Sci. 2010; 135:53–58.

[21] Tian GL, Yang YH, Zhang SP, Miao H, Lu HW, Wang Y, et al Genetic analysis and gene mapping of Papaya ring spot virusresistance in cucumber. Mol Breeding. 2015; 35:110.

[22] Ren Y, Zhang Z, Liu Y, Staub JE, Han Y, Cheng Z, et al An integrated genetic and cyto-genetic map of the cucumber genome. PLoS One. 2009; 4:e5795 10.1371/journal.pone.0005795 19495411 PMC2685989

[23] Wang M, Wen SC, Yan YH. The main antiviral breeding of melon crops (under). Journal of Changjiang Vegetables. 1997; (3):1–5 (in Chinese).

[24] Song ZC, Miao H, Zhang S, Wang Y, Zhang SP, Gu XF. Genetic Analysis and QTL Mapping of Fruit Peduncle Length in Cucumber (Cucumis sativus L.). Plos one. 2016; 11:12 10.1371/journal.pone.0167845 PMC5148027 27936210

[25] Lu HW, Miao H, Tian GL, Wehner TC, Gu XF, Zhang SP. Molecular mapping and candidate gene analysis for yellow fruit flesh in cucumber. Mol Breed. 2015; 35:64.

[26] Chen Z, Zhang MH, Zhou XP, Wu JX. Development and detection application of monoclonal antibodies against Zucchini yellow mosaic virus. Journal of Integrative Agriculture. 2017; 16(1):115–124.

[27] Zvereva A S, Pooggin M M. Silencing and innate immunity in plant defense against viral and non-viral pathogens. Viruses. 2012; 4:2578–2597. 10.3390/v4112578 23202495 PMC3509663

[28] Whitham S, Dinesh-Kumar S P, Choi D, Corr C, Baker B. The product of the tobacco mosaic virus resistance gene N:similarity to toll and the interleukin–1 receptor. Cell. 1994; 78(6):1101–1115. 7923359 10.1016/0092-8674(94)90283-6

[29] Abbink T E, Tjernberg P A, Bol J F. Tobacco mosaic virus helicase domain induces necrosis in N gene-carrying tobacco in the absence of virus replication. Mol Plant Microbe Interact. 1998; 11:1242–1246.

[30] Amano M, Mochizuki A, Kawagoe Y, Iwahori K, Niwa K, Svoboda J, et al High-resolution mapping of zym, a recessive gene for Zucchini yellow mosaic virus resistance in cucumber. Theor Appl Genet. 2013; 126:2983–2993. 10.1007/s00122-013-2187-5 24026172

[31] Zhang HY, Mao AJ, Zhang F, Xu Y, Wang YJ. Mapping of 3 major virus resistant genes in cucumber. J Agric Biotechnol. 2005; 13(6):709–712.

[32] Zhou J. Genetic analysis and gene mapping of Water-melon mosaic virus (WMV) resistence in cucumber. Dissertation, The Chinese Academy of Agricultural Sciences. 2012.

