# Supplementary material for: Inheritance and QTL mapping of cucumber mosaic virus resistance in cucumber (Cucumis Sativus L.)
Source: PLoS One. 2018 Jul 18;13(7):e0200571. doi: 10.1371/journal.pone.0200571 (PMC6051622; doi:10.1371/journal.pone.0200571)
Supplement: S1 Fig — (DOCX) [file pone.0200571.s001.docx]

**
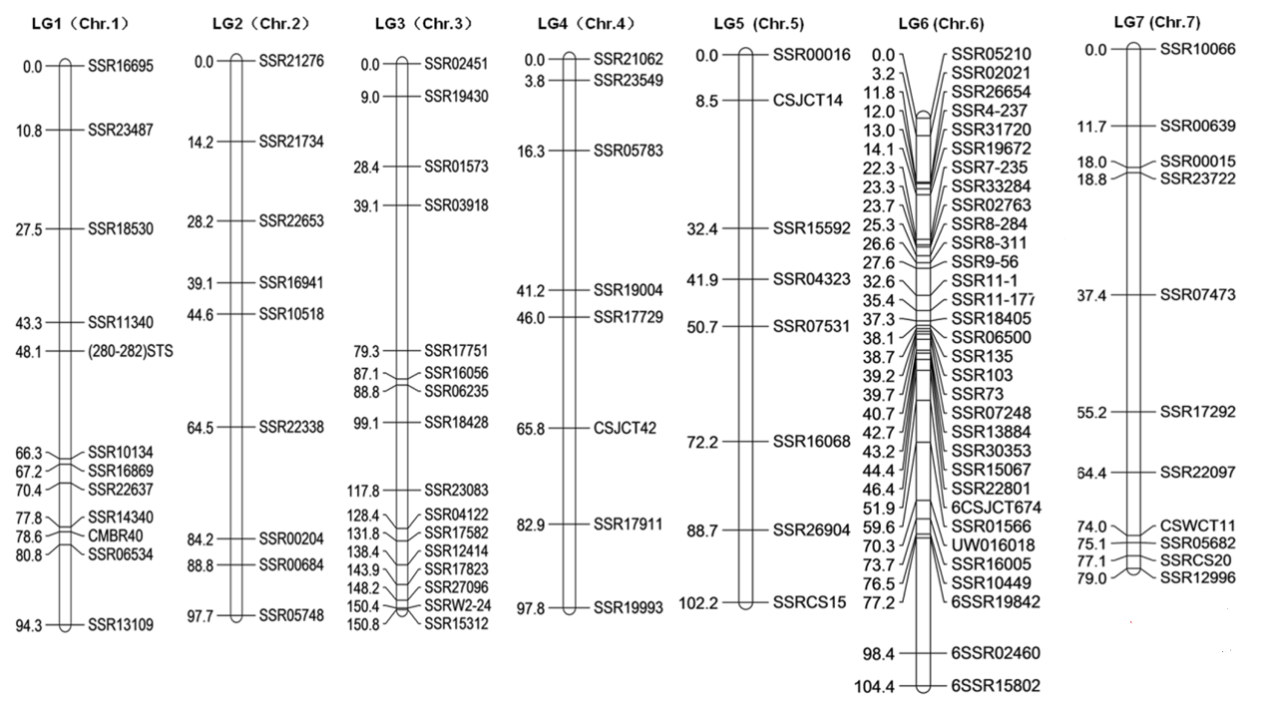
S1 Fig. Construction and QTL Mapping for cucumber mosaic virus (CMV) resistance in a cucumber RIL Population using SSR markers.**
