# Supplementary figures and images for: Inheritance and QTL mapping of cucumber mosaic virus resistance in cucumber (Cucumis Sativus L.)
Source: PLoS One. 2018 Jul 18;13(7):e0200571. doi: 10.1371/journal.pone.0200571 (PMC6051622; doi:10.1371/journal.pone.0200571)

**S2 Fig. Integrated mapping of resistance genes to CMV, WMV, PRSV, and ZYMV in cucumber**


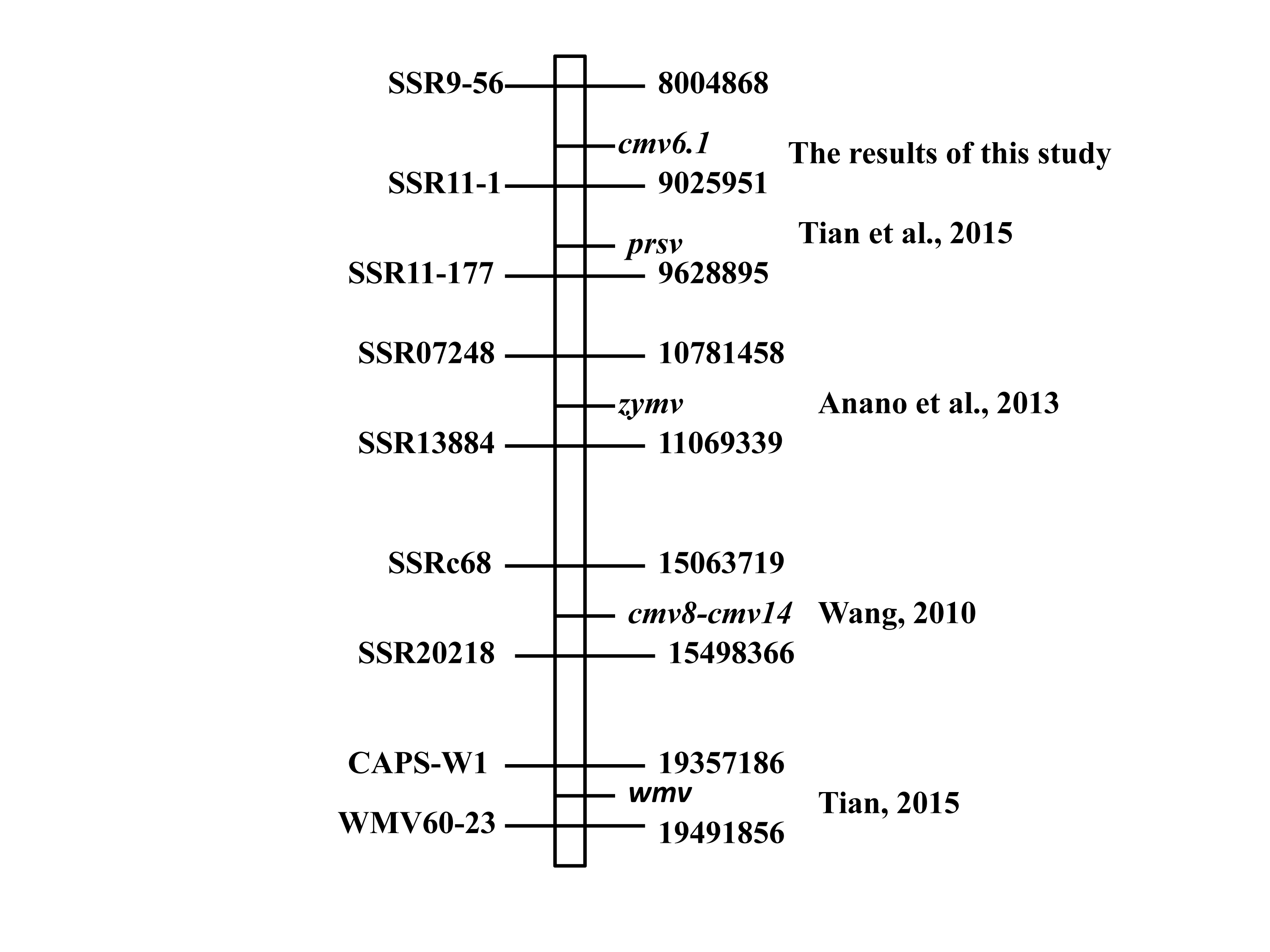

Supplement: S2 Fig — (DOCX) [file pone.0200571.s002.docx]
