# Supplementary material for: Inheritance and QTL mapping of cucumber mosaic virus resistance in cucumber (Cucumis Sativus L.)
Source: PLoS One. 2018 Jul 18;13(7):e0200571. doi: 10.1371/journal.pone.0200571 (PMC6051622; doi:10.1371/journal.pone.0200571)
Supplement: S1 Table — (DOCX) [file pone.0200571.s003.docx]

**S1 Table. Information of 97 SSR markers placed on the 65G×02245 linkage map**

| **Nr** | **Locus** | **Chromosomes**  **(Chr.)** | **Position (cM)** | **Physical position (bp)** | **Forward (5'-3')** | **Reverse (5'-3')** |
| --- | --- | --- | --- | --- | --- | --- |
| 1 | SSR13109 | Chr. 1 | 94.3 | 1318082 | TTTTTCTCCTCGTAAACACATTT | CGTCTTTTCTACTCTTTGGGGA |
| 2 | SSR06534 | Chr. 1 | 80.8 | 4211527 | CATAAAACGGACGTTTCAAAAA | TTTCTTGGAATAGGTGCAATGA |
| 3 | CMBR40 | Chr. 1 | 78.6 | 4553396 | CGACAATCACGGGAGAGTTT | TTGTTGCATCAAACTAACACAATC |
| 4 | SSR14340 | Chr. 1 | 77.8 | 4613278 | TGACCGGAAGTATCATTGGAG | TCCTTTCCTTGTGAATGGAAT |
| 5 | SSR22637 | Chr. 1 | 70.4 | 6818593 | AAAAATCGGTTCAACCCAAC | ACTTCCAACCCAACGACTGT |
| 6 | SSR16869 | Chr. 1 | 67.2 | 7463593 | GAAAACATGAAGGCCGTTGT | CCATTTGTCAGTGCCTTTCA |
| 7 | SSR10134 | Chr. 1 | 66.3 | 8146471 | CCAAAACCAAAAGCAAAATCC | AAATTTGCCAGGAACACCAG |
| 8 | (280-282)STS | Chr. 1 | 48.1 | 13473574 | AACACTCCTGCTTTAACAGCATC | AATGTAATCGTCATTCAGCAGTGT |
| 9 | SSR11340 | Chr. 1 | 43.3 | 15183488 | ATATGTGTGTCGTGTTCCGC | CAGATTTCCGAGAGGGAAAA |
| 10 | SSR18530 | Chr. 1 | 27.5 | 18980946 | CCACAGTCCCACACACAAAG | GGCGTTTTGTGAAGACAGATT |
| 11 | SSR23487 | Chr. 1 | 10.8 | 22019619 | TGTTTCAAGGTGCTGACCTG | CCACAACAACAAAAGAATGTGAA |
| 12 | SSR16695 | Chr. 1 | 0 | 24649281 | CACAATCCCACGAAGAACAA | TGCAATTATGGCAAATCAAAA |
| 13 | SSR05748 | Chr. 2 | 97.7 | 89863 | TGTGGCCTGTGCTAAAATGA | TTTGGAAAAGCTAAAGCCCA |
| 14 | SSR00684 | Chr. 2 | 88.8 | 3255681 | AAGGCCAAAAGACTATGCGA | CATCCCTTGCATCTCCACTT |
| 15 | SSR00204 | Chr. 2 | 84.2 | 4182584 | AACCCTATTTGCACGCATTC | GAGAAACAGCTGGAATTGGG |
| 16 | SSR22338 | Chr. 2 | 64.5 | 8728167 | GGTGGATGAAGAGGGGAAAT | TACTCCTTCCTTCGCCCTTT |
| 17 | SSR10518 | Chr. 2 | 44.6 | 13901019 | TCTAATTCGCTCCGGATGAT | TTGCAGCGAACAATCCTGTA |
| 18 | SSR16941 | Chr. 2 | 39.1 | 15721517 | ATCGGTGGTAGTGGTTACGC | GTGGGGTCCAGAGTTGAAAA |
| 19 | SSR22653 | Chr. 2 | 28.2 | 17526277 | TGAATTTCTTTGGTGGATTCAA | ACCTCCCATGGCAATCTACA |
| 20 | SSR21734 | Chr. 2 | 14.2 | 19046278 | TCCCTCAACGATTCGAGTTT | AAAAGCAAGAAGCAACCCAA |
| 21 | SSR21276 | Chr. 2 | 0 | 21047286 | TCGATGATAAATCCATCACATTTT | CGCTCTTGCCTATCTTGCTT |
| 22 | SSR02451 | Chr. 3 | 0 | 2552417 | TCACCTCCTTCCTCACATCC | GTTCTAGAGGGAGTTCCGGG |
| 23 | SSR19430 | Chr. 3 | 9 | 5572002 | TATGGCGAAGAAGCTTTGCT | AGGGGGATCTTGCCTCTAAA |
| 24 | SSR01573 | Chr. 3 | 28.4 | 8399827 | CGTTAGGCCAAACAAAATTGA | TGCAAACGTTTCTCTAGGCA |
| 25 | SSR03918 | Chr. 3 | 39.1 | 10288739 | TGAGGGTGAGACAACAAATCA | TGTGTTTGACAAAGGAAAGGAA |
| 26 | SSR17751 | Chr. 3 | 79.3 | 16484375 | TCCAACGGAAAATTACAAGGA | TCAATTGTTGGATTCATGTCAA |
| 27 | SSR16056 | Chr. 3 | 87.1 | 20507697 | CATCACGACCCTCTCCATCT | GGGTTTGATAGTGGAGATTATTCA |
| 28 | SSR06235 | Chr. 3 | 88.8 | 22643147 | GGGATGCAGCATGAATGATA | GTGAAAAATTCGGTCAACCC |
| 29 | SSR18428 | Chr. 3 | 99.1 | 26417833 | CCATTCACTTCCTTTCCAGC | TGGTTTCAAGACCACCCTCT |
| 30 | SSR23083 | Chr. 3 | 117.8 | 30347417 | CTTGGGAATTCCACGTCACT | CGTTGAGAAATGCTAATCAATGG |
| 31 | SSR04122 | Chr. 3 | 128.4 | 32322028 | GCCAGGATCAGAAGACGAAG | CTCTTCTCTCTCCCCGGACT |
| 32 | SSR17582 | Chr. 3 | 131.8 | 33164881 | CCGGACGCCAATATATGAAC | GAACCGACAAACCACAAACC |
| 33 | SSR12414 | Chr. 3 | 138.4 | 34574925 | AACGAAAGTAACGCCGTTTG | ACGGAGGAAATCAGAACCCT |
| 34 | SSR17823 | Chr. 3 | 143.9 | 36479408 | TTTTCTTTTTGCAATGGCCT | GATGGGGAAGGGTTTCATTT |
| 35 | SSR27096 | Chr. 3 | 148.2 | 38539946 | GGGACCCAATTCATTGCTTA | TGATACACAAATTCAACTTCACGA |
| 36 | SSRW2-24 | Chr. 3 | 150.4 | 39240126 | CGTTCGAATGAACTCGTGAA | AAGCAACAGGGATGGAACAG |
| 37 | SSR15312 | Chr. 3 | 150.8 | 39401720 | CCCGCTTCTTCTTCCTTCTT | ACTTCTCCGGGAAAATCCAC |
| 38 | SSR21062 | Chr. 4 | 0 | 1862547 | TCACACAACAACTCCATAACACA | GGCCGCGTAGTAGAGTGAAG |
| 39 | SSR23549 | Chr. 4 | 3.8 | 1063659 | TCACCCCCACTTTACTCCTC | AGTCAATCAGTCAGCGCCTT |
| 40 | SSR05783 | Chr. 4 | 16.3 | 3363484 | AAAAGTCCCACAACATATGGAAA | TGGACAAACCAAGTCCCACT |
| 41 | SSR19004 | Chr. 4 | 41.2 | 7480932 | TGAAAAGAACATATGGGGCA | TGCAAACATAACAATTCGATCC |
| 42 | SSR17729 | Chr. 4 | 46 | 8588150 | CTAAAGGGAGGGCTCAATCC | TGATGGGTGTGGGGATAAAT |
| 43 | CSJCT42 | Chr. 4 | 65.8 | 12705199 | GAGAGCCCCACCACCAGTCT | GGATCCATGGCGCCTATAAATACC |
| 44 | SSR17911 | Chr. 4 | 82.9 | 14823864 | TGGAAGAGTGAGAAATGGGC | TGGGAGAAACGTTCTTTATTTTT |
| 45 | SSR19993 | Chr. 4 | 97.8 | 17303911 | ATCCGTGTGATCCTCATCCT | CCACTTGGAAGTTGGAACAAG |
| 46 | SSR00016 | Chr. 5 | 0 | 4616793 | CCCTCTCCCCTCCATGTAAT | TGGGAGTTTGGGTTTACAATC |
| 47 | CSJCT14 | Chr. 5 | 8.5 | 7026115 | TTCCACGTTACATTGGACGA | AGAATTCATGGCCTGCAGAT |
| 48 | SSR15592 | Chr. 5 | 32.4 | 814688 | TTTGATTTCTTGTACCTAAAAAGTAGG | TGCAGTGTCATATGAAGATGGTC |
| 49 | SSR04323 | Chr. 5 | 41.9 | 13208053 | TGGTGGAAAAGAAAAAGGGA | GCTAGGGCACAAGAACGAAG |
| 50 | SSR07531 | Chr. 5 | 50.7 | 17208433 | GAGTCCGTTGGCTTATTTCAA | TCCCTCTTTGTCGGTCCATA |
| 51 | SSR16068 | Chr. 5 | 72.2 | 20995314 | TATAACCCCGTCTTCTCCCC | GGGAAACCCCTTTGACTCTC |
| 52 | SSR26904 | Chr. 5 | 88.7 | 23698084 | CCACCATGTTGTGCTTATCAA | GACCCTTCCAAAAGTAATAAACAA |
| 53 | SSRCS15 | Chr. 5 | 102.2 | 26322919 | ACCGGCATCAAAATAAATGG | GGCGAAAAGGTGAGAAGATT |
| 54 | SSR05210 | Chr. 6 | 0 | 513393 | TTTATGCCATTGTGCGTGAT | TCAAATCAACGTCAACCCAA |
| 55 | SSR02021 | Chr. 6 | 3.2 | 997081 | TAAACATGGCTTCCTCCTCC | CTCTCTTTTCTCACACCCACAG |
| 56 | SSR26654 | Chr. 6 | 11.8 | 4191912 | CACATGAATCGGATTTTGGA | TGCATGATTATGATCTTAGAAAACG |
| 57 | SSR4-237 | Chr. 6 | 12 | 4195306 | CCACAATGTGTTAAAGCACGA | GTTGTTGGCTTTGGTTTGGA |
| 58 | SSR31720 | Chr. 6 | 13 | 4419581 | GACTGTGAATGACGATCTACAGG | CACGAATCATTGCTACAAAAGG |
| 59 | SSR19672 | Chr. 6 | 14.1 | 4943628 | AAGGCAGCAGAAAACTTGGA | CCCTCACTCTCGCTCACTCT |
| 60 | SSR7-235 | Chr. 6 | 22.3 | 6348576 | CCCATCCAAGTTGACTTCACA | ATTGAGGGGACATTTCATCG |
| 61 | SSR33284 | Chr. 6 | 23.3 | 6610818 | TGGGAAATGGATAATTTGGTG | GGCATCCATCATTTCTTTGG |
| 62 | SSR02763 | Chr. 6 | 23.7 | 6444729 | GGGACCTCTGAGCTTTTTCC | AAAACGCAAATGGGTATTCG |
| 63 | SSR8-284 | Chr. 6 | 25.3 | 7299366 | CACAACCACACAATCGATCAG | GGAATGTCATGTCGGCTAAAA |
| 64 | SSR8-311 | Chr. 6 | 26.6 | 7346587 | CAAAGATTGAGAGCAATATTGTGAA | TATGTGCCATAGGCCAACAC |
| 65 | SSR9-56 | Chr. 6 | 27.6 | 7637340 | CAAAACAAGGTAAGGTGTATTGGA | TAAAAGGCAGGACATGCTCA |
| 66 | SSR11-1 | Chr. 6 | 32.6 | 9025951 | ACAAAGCTTCTCCGCAAATG | GGAGGGAAAGGAAGGAGAGA |
| 67 | SSR11-177 | Chr. 6 | 35.4 | 9628895 | CGTGGCATAAAACCACGAAT | CAAATTCAACAAAACCCTACCA |
| 68 | SSR18405 | Chr. 6 | 37.3 | 10051003 | CGCAGGTGCATCTCATGTAA | GACAAACAAGGGGACGAAAA |
| 69 | SSR06500 | Chr. 6 | 38.1 | 10260783 | TGACAAAACACTACCCACCG | AGCAAATTCAACCGTTCATGT |
| 70 | SSR135 | Chr. 7 | 38.7 | 10370639 | GAGGGCAGACCAAAAGAAGA | TGCTTTCTCACGCCCTTTAT |
| 71 | SSR103 | Chr. 8 | 39.2 | 10461122 | GTTGGGTTGTTTTCACCACA | CAAAATTTGAGAACCAGAAAAAGAA |
| 72 | SSR73 | Chr. 9 | 39.7 | 10561951 | AGATATAATTTGCGTGTGTTTGAA | ATGGTCCTTTCCTCCAGGTC |
| 73 | SSR07248 | Chr. 6 | 40.7 | 10781610 | CGATTGGAAAATATCGGCAC | CGAATCGCCTTCAGTTCTTT |
| 74 | SSR13884 | Chr. 6 | 42.7 | 11069339 | GGATAATCCTGATTCCTGTGG | TGTGCAACTGAAAACGAAGC |
| 75 | SSR30353 | Chr. 6 | 43.2 | 11428052 | GGACCATTAACTTCCACCCC | ATGGGATTTGCCTGTAATGC |
| 76 | SSR15067 | Chr. 6 | 44.4 | 13578213 | AACCACTCCCACTTGCCTAA | AAAAATATTCAAACCCAATTTTTGA |
| 77 | SSR22801 | Chr. 6 | 46.4 | 14084714 | GGGTGAGACATAGTTCTGTGTGAA | CTTGACCAAGAGGTCAAAGC |
| 78 | 6CSJCT674 | Chr. 6 | 51.9 | 15918792 | TAGAAAGGAAGGGATGTGATTAGG | ACAGGTGGTTAGAGGTTAGAGCTG |
| 79 | SSR01566 | Chr. 6 | 59.6 | 18090016 | TGGAAAAGCTCAGTTTGTTTGA | TGAGTGCGAACCAACCTACA |
| 80 | SSR60 | Chr.6 | 65.3 | 19491856 | TGCGTATGCCCTTGTATCCT | AAAAGGGACTGGTGTAGACTGC |
| 81 | UW016018 | Chr. 6 | 70.3 | No hits | CCATTTCGACAGTCTCTTCCA | GACGAGGAGACTTGATGGGA |
| 82 | SSR16005 | Chr. 6 | 73.7 | 21343218 | CTCCACGAACCTTCCTTCAC | ATTGTTGGGCTTGGTTCAAT |
| 83 | SSR10449 | Chr. 6 | 76.5 | 21769333 | CACAATTTCCTCCGCTGTTT | CCTTTTCAGCTTCTTCTAATTTCTC |
| 84 | SSR19842 | Chr. 6 | 77.2 | 21813416 | CCCCACCTAAACCTTTCCAT | GTTTGTGCAAAACCCAATCC |
| 85 | SSR02460 | Chr. 6 | 98.4 | 26252541 | CTCAGAAACCCTTCCACCAA | CTGTACCGCGAGGACAGTTT |
| 86 | SSR15802 | Chr. 6 | 104.4 | 27601816 | CAACAAAGCCCTAACAAAAGC | TGCATCTGGTCTCCTTCTTCT |
| 87 | SSR10066 | Chr. 7 | 0 | 1324413 | CCATCAGAACTGTCCATCCG | TTCTGACTGCACAACCAACC |
| 88 | SSR00639 | Chr. 7 | 11.7 | 2401361 | CGAAAACCCCTTTACTTCTCAA | GGCAGCTCCTCCCTCTAAAT |
| 89 | SSR00015 | Chr. 7 | 18 | 3427967 | CCAAACTGGAGGCAAACCT | GAAAAGCACAGCAAAAATCCA |
| 90 | SSR23722 | Chr. 7 | 18.8 | 3424556 | GCATTTGAAAGATGTGTCATCG | AAGGCAAATTAATTAACGAGGG |
| 91 | SSR07473 | Chr. 7 | 37.4 | 6781166 | CGAGTGTGGACATTTTAGAGGG | TTGACTTTCCTCATTCTTTTAATGC |
| 92 | SSR17292 | Chr. 7 | 55.2 | 14006707 | CTCGATTGGAGTCCCTTTGT | CGGACAATTGTGTCAACCAC |
| 93 | SSR22097 | Chr. 7 | 64.4 | 13483521 | ACGGTCATCCGAATTCTCAG | CAACAAACGATCCAACATCG |
| 94 | CSWCT11 | Chr. 7 | 74 | 15794858 | ATAGGCAATATGGCTTCT | CACTTCAATGGAGTTTCG |
| 95 | SSR05682 | Chr. 7 | 75.1 | 16861585 | TGAAGGTTTTTCTCCAGCGT | ATTCGCTCACTTCCGAAAAG |
| 96 | SSRCS20 | Chr. 7 | 77.1 | 17331162 | TTTCTCTCTTTTTCTCTCTTTTTCC | GAGGATTTCCTTCCGATTCC |
| 97 | SSR12996 | Chr. 7 | 79 | 18738941 | TTCTCTCTGACCTTGAAAACAATG | AGCCCATTATCCCACCATCT |
