# Supplementary material for: Inheritance and QTL mapping of cucumber mosaic virus resistance in cucumber (Cucumis Sativus L.)
Source: PLoS One. 2018 Jul 18;13(7):e0200571. doi: 10.1371/journal.pone.0200571 (PMC6051622; doi:10.1371/journal.pone.0200571)
Supplement: S2 Table — Marker genotype designation: a, 65G allele (CMV02245/CMV02245); b, 02245 allele (cmv02245/cmv02245). (DOCX) [file pone.0200571.s004.docx]

**S2 Table. Validity of the marker SSR11-1 tightly linked with *cmv* in 78 cucumber materials.**

| **No** | **Ecotype** | **Phenotype** | **Genotype** | **No** | **Ecotype** | **Phenotype** | **Genotype** |
| --- | --- | --- | --- | --- | --- | --- | --- |
| 1 | Northern China type | R | b | 40 | Northern China type | MR | b |
| 2 | Northern China type | R | b | 41 | Northern China type | R | b |
| 3 | Northern China type | R | b | 42 | Northern China type | R | b |
| 4 | Northern China type | R | b | 43 | Northern China type | R | b |
| 5 | Northern China type | R | b | 44 | Northern China type | R | b |
| 6 | Northern China type | MR | b | 45 | Northern China type | R | b |
| 7 | Northern China type | R | b | 46 | Northern China type | R | b |
| 8 | Northern China type | R | b | 47 | Northern China type | MR | b |
| 9 | Northern China type | R | b | 48 | Northern China type | R | b |
| 10 | Northern China type | R | b | 49 | Northern China type | MR | b |
| 11 | Northern China type | R | b | 50 | Northern China type | S | a |
| 12 | European greenhouse type | **MR** | **a** | 51 | Northern China type | MR | b |
| 13 | European greenhouse type | S | a | 52 | Northern China type | R | b |
| 14 | European greenhouse type | S | a | 53 | Northern China type | R | b |
| 15 | Hybrid of Northern China | **S** | **b** | 54 | European greenhouse type | S | a |
|  | type and European type |  |  |  |  |  |  |
| 16 | Northern China type | HR | b | 55 | European greenhouse type | S | a |
| 17 | Northern China type | R | b | 56 | European greenhouse type | MR | b |
| 18 | Northern China type | R | b | 57 | European greenhouse type | MR | b |
| 19 | Northern China type | R | b | 58 | European greenhouse type | **MR** | **a** |
| 20 | Northern China type | R | b | 59 | European greenhouse type | **MR** | **a** |
| 21 | Northern China type | R | b | 60 | Northern China type | R | b |
| 22 | Northern China type | R | b | 61 | Northern China type | R | b |
| 23 | Northern China type | R | b | 62 | Northern China type | R | b |
| 24 | Northern China type | R | b | 63 | Northern China type | MR | b |
| 25 | Mixture Hybrid of Northern | **S** | **b** | 64 | Northern China type | MR | b |
|  | China type and European type |  |  |  |  |  |  |
| 26 | Northern China type | MR | b | 65 | Northern China type | MR | b |
| 27 | Northern China type | MR | b | 66 | Northern China type | R | b |
| 28 | Northern China type | MR | b | 67 | Northern China type | R | b |
| 29 | Northern China type | MR | b | 68 | Northern China type | R | b |
| 30 | Northern China type | R | b | 69 | Northern China type | R | b |
| 31 | Northern China type | R | b | 70 | Northern China type | R | b |
| 32 | Northern China type | R | b | 71 | The lines derived from Wild type and Northern China type | R | b |
| 33 | Northern China type | R | b | 72 | The lines derived from Wild type and Northern China type | MR | b |
| 34 | Northern China type | R | b | 73 | The lines derived from Wild type and Northern China type | **S** | **b** |
| 35 | Northern China type | R | b | 74 | The lines derived from Wild type and Northern China type | R | b |
| 36 | Northern China type | R | b | 75 | The lines derived from Wild type and Northern China type | MR | b |
| 37 | Northern China type | S | a | 76 | The lines derived from Wild type and Northern China type | R | b |
| 38 | Northern China type | R | b | 77 | The lines derived from Wild type and Northern China type | MR | b |
| 39 | Northern China type | R | b | 78 | The lines derived from Wild type and Northern China type | MR | b |

Marker genotype designation: a, 65G allele (*CMV^02245^*/*CMV^02245^*); b, 02245 allele (*cmv^02245^*/*cmv^02245^*).
